# Surface induced self-organization of comb-like macromolecules

**DOI:** 10.3762/bjnano.2.61

**Published:** 2011-09-12

**Authors:** Konstantin I Popov, Vladimir V Palyulin, Martin Möller, Alexei R Khokhlov, Igor I Potemkin

**Affiliations:** 1 Physics Department, Moscow State University, Moscow 119991, Russian Federation; 2 Institute of Polymer Science, University of Ulm, 89069 Ulm, Germany; 3 Institute of Technical and Macromolecular Chemistry, RWTH Aachen and DWI at the RWTH Aachen e.V., 52056 Aachen, Germany

**Keywords:** comb copolymers, macromolecules, adsorption, self-organization

## Abstract

We present a review of the theoretical and experimental evidence for the peculiar properties of comb copolymers, demonstrating the uniqueness of these materials among other polymer architectures. These special properties include an increase in stiffness upon increasing side-chain length, the spontaneous curvature of adsorbed combs, rod–globule transition, and specific intramolecular self-assembly. We also propose a theory of chemically heterogeneous surface nanopattern formation in ultrathin films of comblike macromolecules containing two different types (A and B) of incompatible side chains (so-called binary combs). Side chains of the binary combs are strongly adsorbed on a surface and segregated with respect to the backbone. The thickness of surface domains formed by the B side chains is controlled by the interaction with the substrate. We predict the stability of direct and inverse disc-, torus- and stripelike nanostructures. Phase diagrams of the film are constructed.

## Introduction

Recent advances in macromolecular synthesis allow precise control over structure and polydispersity of architecturally complex polymers [1–3]. Among these polymers are comb or brush copolymers, i.e., macromolecules which consist of a backbone and attached side chains [4–5]. Originally the interest in comb copolymers was motivated by the desire to achieve liquid-crystalline (LC) ordering of flexible linear macromolecules through the attachment of mesogene side chains and also to enhance the solubility of rigid conjugated polymers such as polyaniline [5–7]. Additional attention to brush copolymers was stimulated by their biological relevance – such an important class of biomolecules as proteoglycans has comblike structure. These molecules are involved in cell signalling and cell surface protection as well as joint lubrication, lung clearance and cartilage stability, cellular matrix integrity [8–13]. Comb copolymers also have unusual viscoelastic properties (super-soft elastomers) [5,14], may form micelles as big as 300 nm [15] and have extremely interesting 2D conformational behavior in the adsorbed state [4]. Brush copolymers with diblock and triblock copolymers as side chains can be used for the creation of well defined organic nanotubes that are soluble in water [16–17].

There are three key methods for the synthesis of graft copolymers [1,5]. The first method involves grafting of previously prepared side chains onto the backbone (the so-called “grafting onto” method). Branch points are obtained by chemical modification of backbone units or by copolymerization with a monomer of the required functionality. The second approach involves the synthesis of active centers along the backbone (the synthesis of macroinitiators) and subsequent growth of side chains from these centers by polymerization (the so-called “grafting from” method). The third approach is termed macromonomer (or “grafting through” method). This method consists of two steps. In the first stage macromonomers (future side chains) are synthesized. Then the copolymerization of the macromonomers and the monomers forming the backbone takes place. Each strategy enables control of different parameters such as grafting density, chemical composition, polymerization degree of side chains and the backbone, polydispersity, etc. Achievement of the desired set of these parameters is quite a complicated task, e.g., due to the steric repulsion of side chains in the case of dense grafting. In some cases a combination of these methods may produce combs which would be otherwise unobtainable.

In this article we pursue two goals. In the first part we give an overview of the peculiar properties of comblike macromolecules, emphasizing the behavior of the macromolecules adsorbed on a surface. In the second part we propose a theory of self-organization of binary combs, i.e., macromolecules with incompatible side chains of types A and B, adsorbed on the surface.

## Results and Discussion

### Properties of comb copolymers

#### Stiffness of macromolecules

One of the most prominent properties of densely grafted comblike macromolecules is their high stiffness induced by strong intermolecular interactions of the side chains. It was suggested that this feature may lead to the creation of systems capable of LC ordering. Such ordering may appear in semidilute solutions of semiflexible polymers if the ratio of the persistence length λ to the diameter of the molecule *D* exceeds some threshold value, λ/*D* > 10 [18–19].

The last couple of years have seen a somewhat revolutionary change in the understanding of the basic properties of single polymer chains [20–21,23]. New theoretical approaches and modeling results indicated that correlations of tangent vectors along a polymer chain are described by a power law instead of exponential decay, even in theta solvent [21] and in the melt [22]. Previously, this fact was also established for chains in a good solvent [24–25]. These findings make the use of the persistence length meaningless as a quantity for the description of the local bending properties. The trajectory of comblike macromolecule (semiflexible cylindrical object) also follows a power law dependence, rather than exponential [23], and thus cannot be correctly described by the persistence length. At first glance, this may bring a two decades long discussion of the scaling properties of the persistence length of comb copolymers to a halt. However, all the inconsistencies are still relevant if as we remind ourselves that the discussion was actually devoted to the bending elasticity, which is described by the bending modulus and not by the persistence length. The latter characterizes orientational correlations in the case of their exponential decay and may not exist, while the bending modulus always does. Nonetheless, in this section we will use the term persistence length for convenience.

Starting with the computer simulation work in the mid 1960s [26], a few experimental and simulation papers, demonstrating the effect of stiffening of the comblike macromolecules with the increase of the side chain length and grafting density, appeared in the 1980s [27–29]. However, only after blob concept was introduced by De Gennes [30], were the first theoretical explanations presented, including a theory by Birshtein et al. [31]. According to this theory, deformation of a comb copolymer with bending less than the diameter of the molecule leads to jumping of the side chains, from concave to the convex side of the “persistence tube”, and the elastic energy increases only when the radius of curvature becomes less than *D*. Hence, it was proposed that the persistence length is on the order of the diameter of the polymer and their ratio does not depend on either the length of the side chains or on their grafting density. Since the approach did not allow an estimation of the coefficients, it was impossible to devise any conclusions about LC ordering in these systems. An alternative theory was developed by Fredrickson [32]. The persistence length of the molecule was estimated by comparison of the free energies of rectilinear and curved brushes, and calculations led to the following result: λ ~ σ^17/8^*M*^15/8^, where *M* is the number of segments in the side chain and σ is the grafting density of the side chains, σ < 1. The expression for the diameter of the tube *D* ~ σ^1/4^*M*^3/4^ agreed with that obtained by Birshtein et al. Thus, λ/*D* ~ σ^15/8^*M*^9/8^ and was much higher than unity at σ >> *M*^−3/5^, i.e., according to the theory of Fredrickson, nematic ordering is possible. Numerous later theoretical and experimental works [33–43] have not reached a consensus over the scaling of the persistence length with the diameter of comb molecules, nor about the possibility of LC ordering (for detailed discussion, see another review [4]).

Notwithstanding the difficulties, all the studies indicated a substantial increase in the stiffness for the backbone and the side chains. For the discrepancies Binder et al. [23] offered two possible explanations. First, they may arise because of the use of the persistence length in the experiment, while the use of alternative measures of rigidity may lead to consistent results between modeling and experiment. Second, the scaling regime discussed by theoreticians is not attainable in experiments, because the length of the side chains is rather short (around 100 monomer units or less) [23,44–45]. As a conclusion of this section, we hope that further accurate studies and comparison of theory and experiment in light of new discoveries [20–23] may resolve an almost two decade old contradiction [31–32].

#### Combs with complex chemical structure

**Figure 1:**
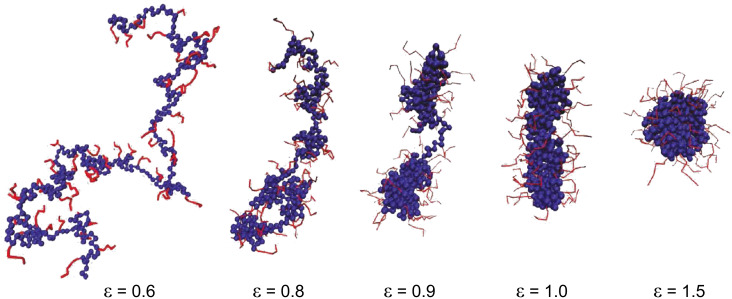
Illustration of the transition from a wormlike structure through a cylindrical micelle down to a spherical micelle, with decreasing solvent quality for the backbone. To make the structure of the backbone clearly visible, the side chains are only drawn as thin lines. Reprinted with permission from Kosovan, P.; Kuldova, J.; Limpouchova, Z.; Prochazka, K.; Zhulina, E. B.; Borisov, O. V. *Macromolecules* **2009,** *42,* 6748–6760. Copyright 2009 American Chemical Society.

After revealing the properties of combs with homopolymer side chains, the next logical step is an increase in the complexity of the chemical structure of the system under consideration. If more than one type of monomer unit is introduced in the comb copolymer a whole range of questions arises, the most interesting being: What types of intramolecular aggregation may occur in solution? The case of a hydrophobic backbone with hydrophilic side chains has revealed quite interesting behavior [46–48]. Both scaling theory [46–47] and simulations [47–48] predicted that collapse of the main chain with increase of hydrophobicity in densely grafted combs would lead to formation of a necklace of intramolecular starlike micelles, with hydrophobic corelike domains connected by extended bridges and hydrophilic corona formed by the grafts (Figure 1). Scaling analysis reveals that the formation of finite-size intramolecular micelles happens only in a narrow range of interaction parameters near the transition point. A similar pearl-necklace structure was detected experimentally for core–shell cylindrical polymer brushes with a solvophobic inner (core) block and a solvophilic outer (shell) block in selective solvents [49].

Combs with two types of incompatible side chains (binary brushes) obviously represent quite intriguing objects due to the potential for intrachain segregation leading to Janus-like structure. Theoretical analysis, within the Flory–Huggins approach, of the intrachain segregation [50–51] demonstrated that, as the quality of the solvent worsens, segregation occurs at lower values of the parameter χ_AB_. In the case of a poor solvent, the segregation condition χ_AB_*M* ~ 1 qualitatively corresponds to the spinodal conditions for microphase segregation in melts of diblock copolymers [52]. Calculation of the free energy for a comblike copolymer with complete segregation of side chains showed that a molecule can spontaneously curve. A simulation study [53] confirmed the existence of spontaneously curved conformations under certain conditions, but failed to find the regime of complete separation into two distinct domains.

Further studies were done by Binder et al. [44–45] for combs with high grafting density (bottle-brushes). They suggested that separation in comb copolymers with two types of side chains is a phase transition in a quasi one-dimensional object. Hence, an ordered state is impossible according to Landau theorem [54]. In all the cases under consideration (poor, theta, and good solvents, and various forces of interaction between units of side chains of various types), correlations along the backbone rapidly decayed [45]. The extrapolation of the correlation length to *T* → 0 showed that, even in this case, there was no long-range ordering. Moreover, Binder et al. mention that at finite *M* values, the cross section will look like a butterfly rather than a circle, as this ensures the smaller number of contacts between A and B units. Segregation will proceed according to the Janus cylinder type only if the energy of attraction between equivalent units is much higher than the energy of repulsion between units of different kinds. As in [53], it was shown that if the solvent is selective for A and B chains, spontaneous curvature of the molecule will occur.

Recent data obtained from off-lattice molecular dynamics simulations [55] suggest that pearl-necklace type separation is also possible in binary bottle-brushes. This type of separation induced by the decrease in the solvent strength was also predicted for combs with one type of side chain through scaling [56], self-consistent field theories [57] and computer simulations [58]. Such a structure was shown to be stable for intermediate and small values of grafting density.

Janus cylinders were experimentally obtained in a rather different way by dissolution of a microscopically separated polymer melt of triblock copolymers [59–60]. At first, a phase is obtained, where the middle block forms thin cylinders on the border between lamellae formed by the outer blocks. Subsequently, the chains in the middle block are cross-linked, and in the last stage, the melt is dissolved. Each comb in the solution consists of a backbone formed by cross-linked middle blocks, and end blocks remain in segregated state after dissolution.

#### Microphase separation

Microphase separation in block copolymer melts has attracted significant attention over the past several decades [61–64] because it produces a fascinating set of ordered nanostructures, which are envisioned to become a core solution of many applications [65–66]. At first, researchers concentrated on the detailed study of self-organization in melts of diblock copolymers [52,67–68]. Later on the interest in the search for novel morphologies shifted to the consideration of copolymers with complex architectures [69–70], nanoparticles imbedded in block copolymer matrices [71], etc.

Before the successful controlled synthesis of graft copolymers [1–2], microphase separation in combs was modelled by theoreticians mostly within the weak segregation theory (WST) approach [52,72]. Spinodals of microphase separation were calculated for melts of comb copolymer in which the backbone and the side chains were chemically different units [73]. It was shown that the transition from a homogeneous to an ordered state is determined by the parameters of the repeating unit, each unit consisting of a spacer between the adjacent branch points, and of the side chain. An increase in the number of these elements ceases to influence the spinodal curves after it exceeds about 20 units. The spinodals were constructed for two types of branching point distributions: Regular and random [74]. The authors found that spinodals of microphase separation for different distributions converge to different limiting curves while having the same chemical composition. A theory of microphase separation in melts of double comblike copolymers was developed in [75–76]. This kind of comb contains two different types of side chains attached to common branch points in a pairwise fashion (A and B side chains are attached to a common unit of the backbone). In [75], copolymers with a regular distribution of branch points were examined. The behavior of a spinodal as a function of the number of repeating units showed two characteristic types. For one combination of the parameters, abrupt changes in the wave vector characterizing the instability of the homogeneous state were discovered. These changes are specific for systems with two characteristic length scales corresponding to the lengths of the backbone and side chains. The existence of these abrupt changes implies that systems with two different scales can form periodic microstructures that are of great interest for potential applications. This so-called two-scale instability was discovered first in comb-coil copolymers [77–78] which consist of comb-like and linear blocks and represent an example of high architectural complexity for graft copolymers. The effect of the distribution of branch points of the side chains on the spinodals of microphase segregation in melts of double comb-like copolymers, was considered in terms of the weak segregation theory by comparison of regular, random and gradient distributions [76]. It was demonstrated that an increase in the nonuniformity of the distribution of the side chains widens the stability region of the microphases. Abrupt changes of the wave vector of the microstructure are also possible for the nonuniform distribution.

#### Liquid-crystalline side chains

Multiscale ordering, which was discussed in the previous subsection, can be achieved in a different way, namely through comb copolymers with liquid crystalline side chains (SCLC). The self-assembly of such copolymers with LC chains is different from that of their flexible counterparts, due to the combined possibility of microphase separation with LC ordering of stiff segments. For example, already linear rod–coil copolymers exhibit such non-trivial morphologies as arrowhead, zigzag, wavy lamellar and smectic bilayers [79–80]. For more details, see recent review [81]. LC phase transition temperatures were found to be close to the homopolymer case. However, in several examples stabilization of liquid-crystalline and microphases influenced each other. Zhang and Hammond achieved the stabilization of the smectic phase by lamellar phase formation [82]. Influence of LC transition on microphase segregation was observed in [83] where the authors demonstrated that the transition from a body-centered cubic morphology to a hexagonal one was stimulated by an isotropic–nematic transition. In another case, a mixed lamellae/cylinder phase transformed into a pure lamellar one as a result of the loss of LC ordering [84].

The first theories to described the SCLC copolymers appeared in the 1980s, on the basis of lattice models [85–86], as a response to the achievements in synthesis [87]. Subsequently, a more rigorous approach, with inclusion of the Maier–Saupe form of interaction, was suggested by Warner and Wang [88]. Spinodals of the microphase separation of a SCLC copolymer with LC groups, attached to the backbone through the flexible spacers, were calculated in [89].

**Figure 2:**
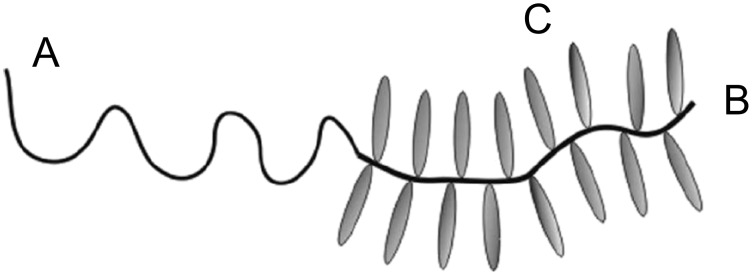
Schematic representation of coil–LC comb copolymer.

From a practical point of view coil–LC comb copolymers, i.e., copolymers which contain a coil block and a block with LC side chains (Figure 2), are more interesting than SCLC copolymers without a coil block. The main reason is simple: Microphase separation in the latter case between the backbone and the LC side chains is achievable, but the period of the microstructures is strongly limited by the length of the spacer between adjacent LC side chains. Coil-comb architecture allows better control of this parameter and the symmetry of the phases, and thus attracts more attention from both experimentalists [82,90–93] and theoreticians [94–96].

**Figure 3:**
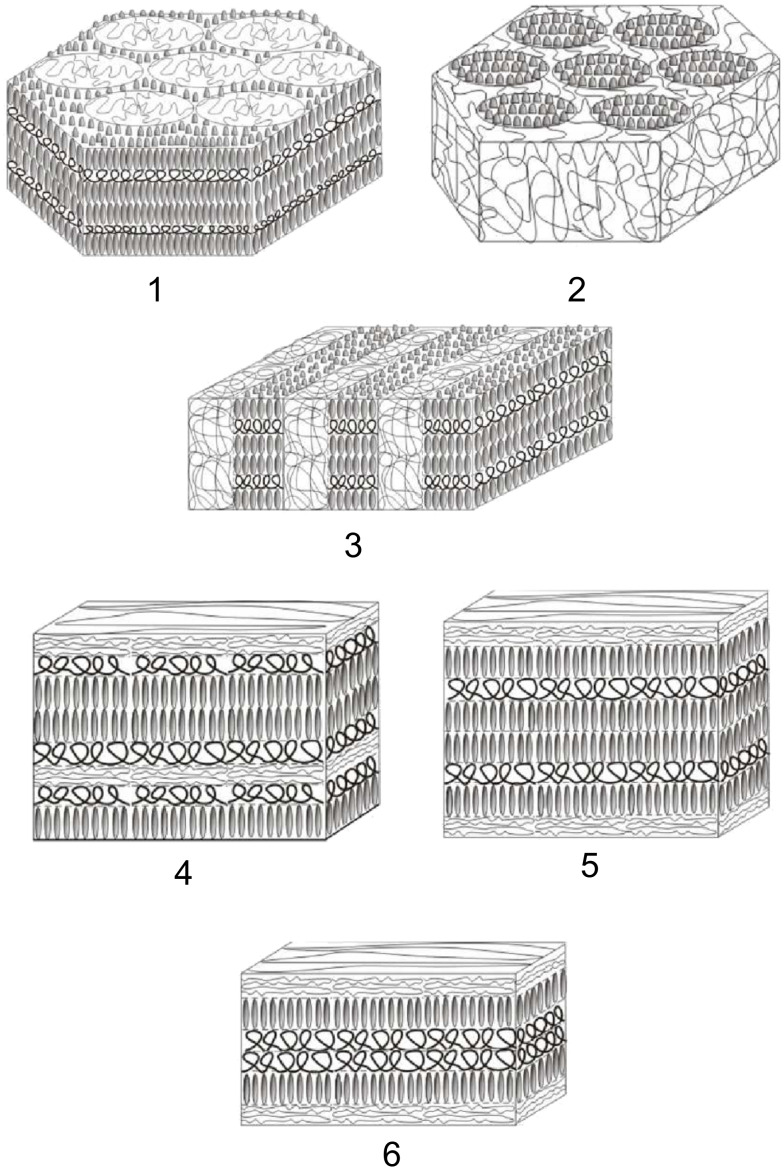
Stable morphologies in coil–LC comb copolymer melts.

In the first of the theoretical publications concerning coil–LC comb copolymers [94], the authors plotted phase diagrams by comparison of the free energies of homogeneous, lamellar, cylindrical and spherical morphologies. The free energy was calculated by summation of the bending energy of the wormlike chain of the backbone, the Maier–Saupe contribution for LC ordering of side LC chains, the stretching energy of the amorphous block, the surface tension and the mixing Flory–Huggins contributions. Later studies [95–96] used SCFT and strong segregation theories. In particular, it was found that, for the probed parameter space, microphase separation is necessary in order to achieve the orientational ordering [95]. The structure of lamellar and cylindrical phases was considered in more detail in [96]. Stability regions of two different cylindrical and four different types of lamellar phases were found (Figure 3). Conditions for stability of each structure can be summarized as follows [96]:

1. Amorphous cylinders: Long macromolecules; high fraction of the B and C (LC) units; any values of the surface tension coefficients satisfying the strong segregation conditions.
2. Liquid crystalline cylinders: Long macromolecules; high fraction of the A units and small enough fraction of the liquid crystalline units; any values of the surface tension coefficients satisfying the strong segregation conditions.
3. A
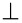
B lamellae: Long macromolecules; the A block has to be a bit longer than the B block; small enough fraction of the liquid crystalline units; any values of the surface tension coefficients satisfying the strong segregation conditions.
4. BAB lamellae: Short enough macromolecules; high fraction of the A units; small enough fraction of the liquid crystalline units; high values of the surface tension coefficient γ*_AC_*.
5. ABB lamellae: Short enough macromolecules; high fraction of the A units; high enough fraction of the liquid crystalline units.
6. ABA lamellae: Short enough macromolecules; high fraction of the A units; high fraction of the liquid crystalline units.

Theoretical predictions [96] were consistent with experimental results [93] for the transition between amorphous cylinders and A
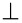
B lamellar phases. In the experiment [93], wedge-shaped molecules with sulfonic group at the tip have been incorporated into a poly(2-vinylpyridine)-*block*-poly(ethylene oxide) (P2VP-*b*-PEO) diblock copolymer by proton transfer at different degrees of neutralization. Then scanning force microscopy (SFM) and X-ray studies were applied to assess the morphology. The agreement was found to be especially good for the diameters of cylindrical domains in the amorphous cylindrical phase, for different degrees of neutralization.

The evident tendency of researcher to study more complex systems is represented by the use of mean-field theory [97] to investigate diblock copolymers with both blocks containing mesogene groups. It was predicted that the lamellar phase has an unusual nonlinear soft elastic response due to the rotation of the LC groups.

### Comblike macromolecules adsorbed on a flat surface

The physical behavior of adsorbed comb copolymers is much better understood, through the direct visualization of the molecules by SFM [98]. SFM allows the determination of the conformational characteristics such as contour length, comb width, backbone curvature, radius of gyration, etc. After adsorption the macromolecules can adopt many different conformations including globular, coil- and rod-like. The physical phenomena behind this complex behavior are described in the following few subsections.

#### Bending modulus

In comparison with the macromolecules in solution, strong adsorption makes most of the side chains two-dimensional (2D). This leads to a large stretching of the 2D chains, *D* ~ *M*, in contrast to a weaker exponent for the 3D case, *D* ~ *M*^3/4^. Theoretical calculations for the 2D comb predicted a dependence of the bending modulus on the number of segments in the side chain as *M*^3^, both for symmetric or asymmetric distributions of side chains relative to the backbone [99]. For combs made of polyhydroxyethyl methacrylate backbone (*N* = 2150 ± 100) and PBA side chains (polymerization degree *n* = (12 ± 1)−(140 ± 12)) adsorbed from good solvent on mica, SFM results for the bending modulus are approximated by the exponents ν = 2.7 ± 0.2 [100], which is close to the theoretical prediction of 3. The difference between the experimental and theoretical results may be attributed to the incomplete adsorption of side chains: Part of the chains forms a 3D brush atop a 2D monolayer. Therefore, a “2.5”-dimensional model including two objects, a 2D monolayer and a 3D semi-cylinder, can be used to approximate the shape of the adsorbed brush. Within this model, the bending modulus of the adsorbed brush in a poor solvent can be approximated as follows [101]:

[1]
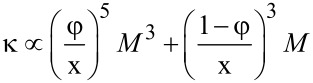


where φ is the fraction of adsorbed chains and parameter *x* ≤ 1 is the ratio of the brush length *L* to the contour length of the backbone *aN*. Parameters φ and *x* depend on the energy of attraction to the surface. Equation 1 shows that if the bending modulus is approximated solely by the power-law function κ ~ *aM*^ν^, then, depending on the strength of adsorption, the exponent will be in the range 2–3, in agreement with the above reported experimental value [100].

Interactions between the 2D side chains induce a very strong force, elongating the backbone. Furthermore, if the length of the side chains exceeds some threshold value, the brushes undergo self-scission because of the breaking of the covalent bonds in the main chain [100,102].

#### Spontaneous curvature

If the distribution of 2D side chains is “frozen” and asymmetric, then the comb molecule will form curved and snakelike conformations [99,103–104]: The higher the asymmetry, the bigger the curvature. It is surprising that if one allows the possibility of the side chains “jumping” from one side to the other, rectilinear conformation of the brush with symmetric distribution of the side chains will not be reconstructed, despite a penalty in the mixing entropy. It was found that the self-equilibration of 2D brushes results in their curvature [105–109]; the explanation was provided in the theoretical papers [107–109]. The free energy of the curved conformation is smaller due to the decrease in the extension of the side chains under their asymmetric distribution. On the convex side of the brush, the extension drops due to enlargement of accessible space, while on the concave side it decreases due to a reduction in the number of side chains. Further computer simulation [110] and theoretical [111] studies confirmed the existence of the spontaneous curvature of adsorbed comb macromolecules. Similar results were obtained for brush membranes within the self-consistent field approximation [112]. In addition, all theories predict the existence of a small barrier for the bending free energy.

#### Rod–globule transition

Brush molecules adsorbed at the water/air surface with the Langmuir–Blodgett technique have shown an ability to undergo transition from straight (rodlike) to globular conformation [98,105,113]. For potential applications there exists the interesting possibility to govern this process either by lateral compression-expansion of the film [105] or by a change of the spreading parameter by admixing of an organic solvent [113]. The character of rod-to-globule transition depends on the length of the side chains: Discontinuous (first order) transition was observed and quantified for brushes with long side chains [105].

Recently, a series of works [114–118] has revealed a new way to change the conformation of brushes adsorbed on solid substrates. Relative changes in composition of water/ethanol vapors lead to reversible transformations from the extended to the compact globular conformation both for isolated molecules [114] and dense films [118].

Interesting results were obtained for the critical exponent ν of the end-to-end distance of the adsorbed brushes [117–118]. Adsorption of isolated combs of poly(butanoate-ethyl methacrilate)-*graft*-poly(n-butyl acrylate) were studied in [117]. Immediately after adsorption, a value of ν = 0.77 was measured, which is close to the 2D statistics of a polymer chain with excluded volume interactions. This quantity dropped to 0.53 after a collapse–reexpansion cycle. The proposed explanation was the following: Initially after deposition from a good solvent the molecule adopts the conformation with a symmetric distribution of the side chains (left–right distribution), which is kinetically trapped. After transition to a globular state and reexpansion, the side chains have the possibility to rearrange thus forming an asymmetric distribution. The asymmetric distribution is thermodynamically more favorable [104] and leads to the snakelike structure with ν ~ 0.5.

In the case of the dense brush monolayer, the exponent ν ~ 0.75 practically does not change after the collapse–reexpansion cycles [118]. The monolayer was prepared by LB technique and transferred on mica. In the LB monolayer, each individual chain saves the conformation of the single 2D molecule, hence ν ~ 0.75. After the collapse–reexpansion cycle, the exponent is slightly smaller, ν = 0.73, but still larger than that for the isolated molecules. One possible explanation is the idea of “memory” of the intermediate conformation in the collapsed state. Another explanation takes into account the balance between the surface energy of 3D aggregate and the stretching free energy of combs in the film [118].

#### Tadpole conformation and the idea of a molecular motor

Quite an important parameter for the rod–globule transition is the grafting density of the side chains. A more densely grafted brush becomes globular much earlier upon an increase in the surface pressure. A good example is provided in experiments with a gradient in the grafting density along the backbone of the brush [119]. An increase in the surface pressure led to rod–globule transition at the end of the brush with a higher grafting density, thus leading to tadpole-like form of the comb molecules. This kind of molecule may serve as a molecular motor by analogy with the directional movement of diblock copolymers [120]. Computer simulations demonstrated that diblock copolymer adsorbed on a striped surface can shift preferentially in one direction if one of the blocks undergoes periodic collapse and readsorption [120]. In the case of combs, a difference in the desorption properties between the sparsely and densely grafted ends may have the same effect on the movement [4,120].

### Nanostructures in monolayers of binary comb copolymers

Thin films of block copolymers have attracted considerable attention as a convenient material for the preparation of heterogeneous surfaces. The proximity of macroscopic phase boundaries affects the orientation of the nanodomains as well as the film structure. Parameters, which govern the orientation, are the interfacial energy of the boundaries [121–122] and the film thickness [123]. In the case of lamellae-forming symmetric diblock copolymers, the perpendicular orientation of the lamellae was found to be stable if the polymer had a high molar mass [123–124] or if none of the blocks had a strong affinity towards the substrate or the air [125]. Otherwise, the lamellae have a parallel orientation with respect to the substrate [125]. Many other factors such as an electric field [122], or competition between the non-lamellar bulk morphology and the affinity of the blocks to the surface [126], or chemically patterned substrates [127–128] may also have a strong influence on the orientation of the diblock copolymer domains.

A chemically heterogeneous surface pattern can reliably be generated from ultrathin films with thickness much smaller than the equilibrium period of the bulk morphology. For instance, this can be obtained by the adsorption of a polystyrene-*block*-poly(2,4-vinylpyridine) diblock copolymer (PS-*b*-P2,4VP) on mica from a non-selective dilute solution [129–133]. The P2,4VP-block is strongly adsorbed and forms approximately a monolayer; the other (PS)-block is incompatible with the air, substrate and P2,4VP-block. In order to reduce the number of unfavorable contacts, PS aggregates into clusters which are stable over a very wide range of block lengths [134]. A different situation can be observed when the stickiness of one of the blocks is variable (for example, poly(ethylene oxide)-*block*-poly(2-vinylpyridine, PEO-*b*-P2VP)). Variation of the stickiness can result in surface nanopattern formation [135]. A theoretical study of ultrathin films of diblock copolymers with varying stickiness of one of the blocks shows that surface nanopatterns with disc-, stripe- and holelike structures can be obtained. If both blocks can be partially desorbed and one of them can spread atop the other block [136], a wider set of morphologies appears: Stripes, discs, holes, bilayers, substratephobic stripes and discs, etc. [136].

Binary comb copolymers comprising incompatible side chains of A and B types attached to a common backbone, may also be used as building blocks to form novel nanopatterns. In our previous work we demonstrated that the conformation of the adsorbed binary comb molecule has a controlled spontaneous curvature [137]. Thus, repulsion of the side chains of different type, on the one hand, and connectivity of the side chains by the backbone, on the other hand, lead to the formation of intramolecular structures. The goal of this section is to study the self-assembly of ultrathin films of binary comb copolymers within a strong segregation approach.

#### Model of ultrathin film of binary combs

**Figure 4:**
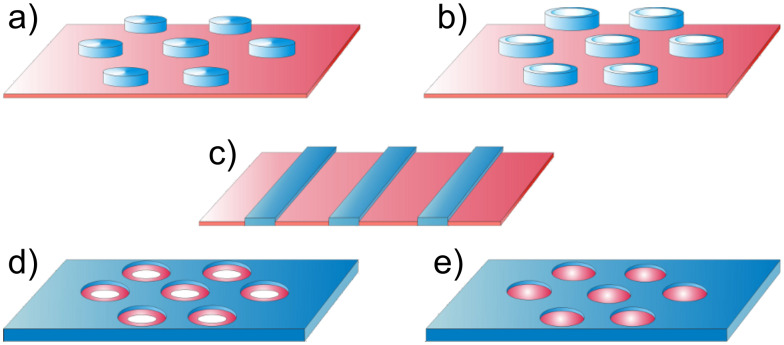
Schematic representation of some of the possible nanostructures formed by binary combs with strongly adsorbed A side chains (red) and partially desorbed B chains (blue): (a) Disc-shaped micelles ordered with the symmetry of a hexagonal lattice (HEX); (b) Torus-shaped micelles ordered with HEX symmetry; (c) parallel stripes; (d) Inverse torus-shaped micelles ordered with HEX symmetry; (e) inverse disc-shaped micelles (“holes”) also ordered with HEX symmetry.

As in [135], we study here a dry, ultrathin film of binary comb copolymers, which were obtained by adsorption of the blocks on a flat surface from a dilute solution. The usual procedure for the preparation of these films involves immersion of the substrate into the solution and pulling it out. Hence, we assume that the overall number of adsorbed macromolecules is constant, but the surface area is still larger than the total area covered with polymer segments. Thus we may analyze the thermodynamically stable morphologies through the variation of the spreading parameter (film area). Each brush molecule contains two types (A and B) of incompatible, flexible side chains. It was assumed that the sequence of grafting points of A and B chains is regularly alternating. Let us denote by *N*, *M*_A_, and *M*_B_ the number of segments in the backbone, A and B side chains, respectively; *N,M*_A_,*M*_B_ >> 1. It was assumed that the linear size of each segment of the brush is equal to *a*. We studied densely grafted combs, i.e., the side chains are attached to each segment of the backbone and their number is equal to *N*. The fraction of the side chains of type B is denoted by β = *N*_B_/*N*, where *N*_B_ is the total number of side chains of type B. The side chains of type A are strongly adsorbed on the surface and form a layer of the thickness *a*. This allows us to consider the latter as a two dimensional object with densely packed units, as it minimizes the number of unfavorable contacts between A units and the air. The B chains are strongly incompatible with the A side chains and may adopt both the two-dimensional (adsorbed) conformation as well as partially desorbed (“shrunken”) one. Adsorption and desorption of the B chains is controlled by the interactions with the substrate. The sum of contributions from the interfacial interactions and the entropic elasticity of the side chains determines whether the lateral segregation is possible. The following structures are involved in the analysis of thermodynamic stability (Figure 4): Disclike structure (a) and the structure of the inverse discs (e); hexagonally packed micelles with a toruslike core formed by partially desorbed B chains (b); parallel stripes (c); and hexagonally packed inverse toruslike “holes” formed by strongly adsorbed A chains in the matrix of partially desorbed B chains (d). The analysis is performed within the strong segregation approximation [68].

#### Discs

**Figure 5:**
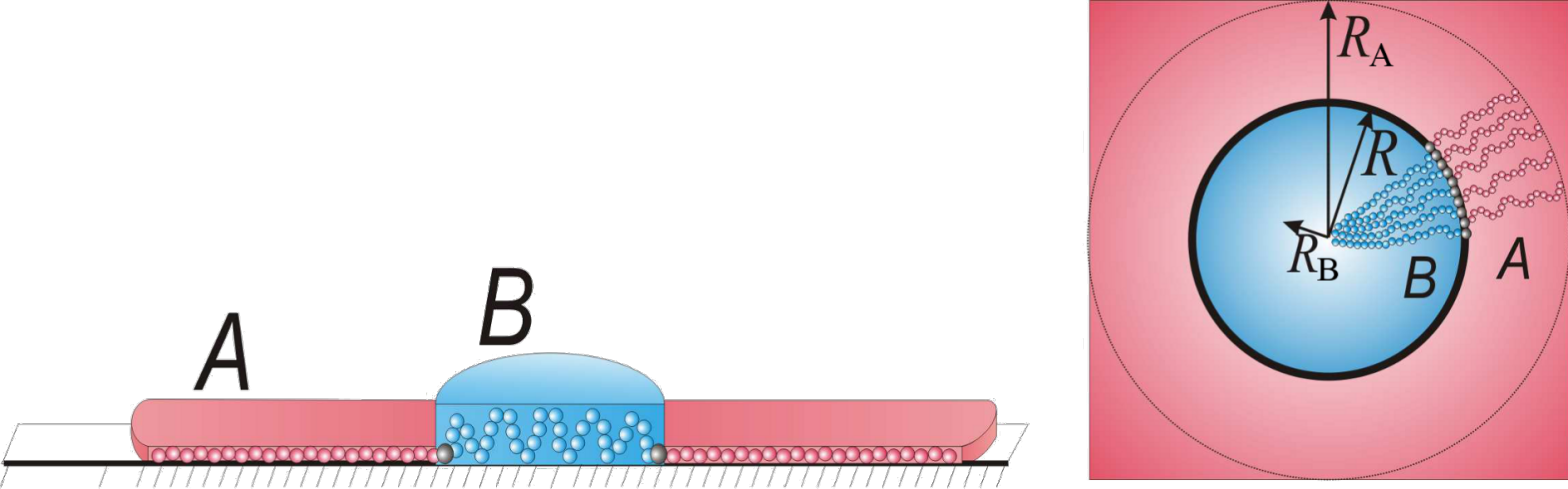
Schematic representation of the disc-shaped structure.

In the model of the disclike micelle, we assumed that the core is formed by the side chains of type B and has a disc shape of radius 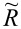 and the thickness 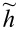 (Figure 5). We studied the regime of weak desorption of the B chains where the end-to-end distance of them is considerably larger than the thickness of the core, *a* ≤ 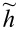 << 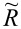. Side chains of the A type are strongly adsorbed and form the shell of the micelle with outer radius 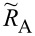 and thickness *a*.

The free energy of the micelle can be written as a sum of four contributions:

[2]
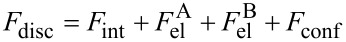


The first term, *F_int_*, is the interfacial energy:

[3]
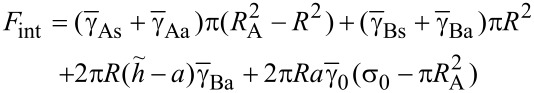


Here, 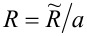 and 
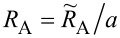
. The first term in Equation 3 is the energy of interactions of the A side chains with the substrate and the air; 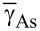 and 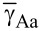 are the corresponding surface tension coefficients. The next two terms describe interactions of B chains with the substrate and the air. The last term in Equation 3 corresponds to the energy of the substrate/air surface; 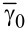 is the substrate/air surface tension coefficient; σ_0_ is the area of the substrate divided by the number of micelles on the substrate. This contribution describes spreading of B chains on the surface: It is not a constant as it would be in the case of the fixed film area. Each micelle comprises *Q* macromolecules. The condition of the dense packing of monomer units in the core and in the shell of the micelle can be written as:

[4]
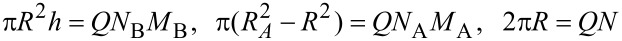


Here, the volume per monomer unit is assumed to be equal to the cube of the segment length. The last condition corresponds to the fact that the backbone of each particular brush molecule is almost fully stretched. Using the above conditions, the interfacial energy per macromolecule (divided by a constant *N*) can be written in the following form:

[5]
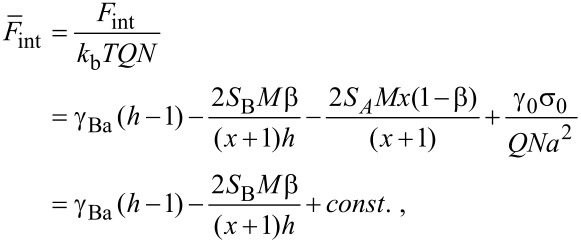


where 
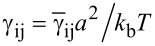
 are the dimensionless surface tension coefficients; *x* = *M*_A_/*M*_B_, (*x* ≥ 1), and *M* = (*M*_A_ + *M*_B_)/2. *S*_A_ = (γ_0_ − γ_Aa_ − γ_As_) and *S*_B_ = (γ_0_ − γ_Ba_ − γ_Bs_) are dimensionless spreading parameters, which control the stickiness of the side chains. In our case, *S*_A_ is fixed, positive and should be fairly large to provide a monomer thick layer for A chains. We considered only variation of the stickiness of B chains. In the system with a fixed number of chains, parameter σ_0_γ_0_/*NQ* (the area of the substrate divided by the number of chains) is constant for all the nanostructures examined, and therefore, it can be omitted.

Now let us calculate the elastic free energy of the side chains (terms 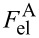 and 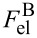 in Equation 2) which can be calculated by analogy with [137]. For the shell (A chains) we supposed that all chains are equally stretched and their ends are located at the outer boundary (Figure 5). The elastic free energy can be written as:

[6]
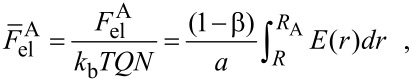


where *E*(*r*) = *dr*/*ds* is the local stretching of the side chain, which depends on radial coordinate *r*. The expression for *E*(*r*) can be calculated using the differential form for the dense packing condition of the monomer units in the ring of width *dr*: 2*πrdr* = *adsQN_A_*. Therefore, taking into account the space filling condition Equation 4, we get:

[7]
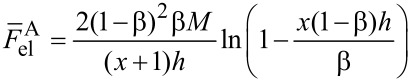


To calculate the elastic free energy of B side chains, the radial distribution of the free ends has to be taken into account. This term can be approximated by the one obtained for the case of cylindrical micelles in the bulk [68]:

[8]
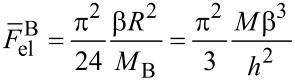


Conformational entropy loss due to adsorption of the side chains comprises contributions from A and B units. The free energy of the B chains can be calculated as that of the chains placed in a slit of thickness *h*. Owing to the condition *R* >> *h*, we can use the so-called ground-state approximation [138], where the energy per monomer unit is the minimum eigenvalue λ of the differential equation:

[9]
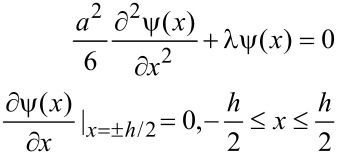


The boundary conditions are taken to satisfy the requirement of a constant density of monomer units inside the slits. The solution of Equation 9 has to be symmetric with respect to the coordinates origin (the middle of the slit), i.e., 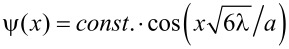. Thus, the confinement free energy per chain takes the following form:

[10]
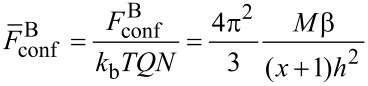


For A side chains the confinement free energy is constant for all the structures considered and therefore can be omitted. From Equation 5, Equation 7, Equation 8, and Equation 10, the total free energy of the disc-like micelle (per one molecule), Equation 2, can be written as:

[11]
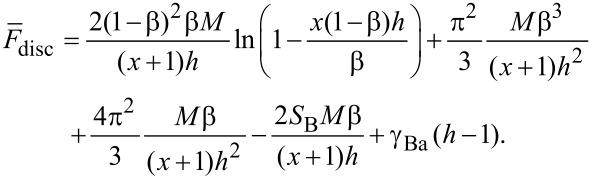


The equilibrium value of the free energy is calculated by minimization with respect to the thickness *h*.

#### Tori

**Figure 6:**
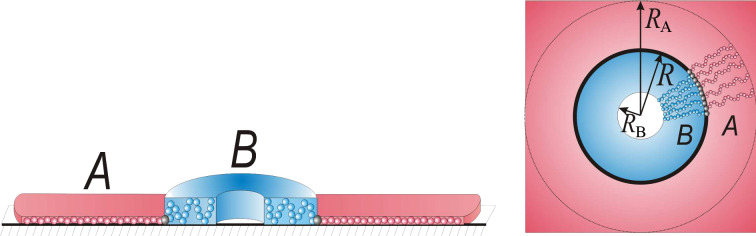
Schematic representation of torus-shaped structure.

The toruslike micelle has a dense, torus-shaped core of thickness 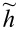 and of radii 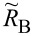 and 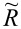 formed by the B side chains. A smoothed profile of the core can be approximated by a step-like shape (Figure 6) if the width of the torus, 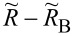, is much larger than the thickness 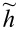. Strongly adsorbed A side chains occupy a ring of thickness *a* and of outer radius 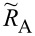.

The general form of the free energy can be described by Equation 2. In the case of torus-like micelles the condition for the dense packing of monomer units in the core and in the shell of the micelle can be written as:

[12]
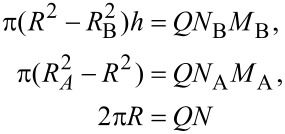


Interaction contribution *F*_int_ takes the form:

[13]
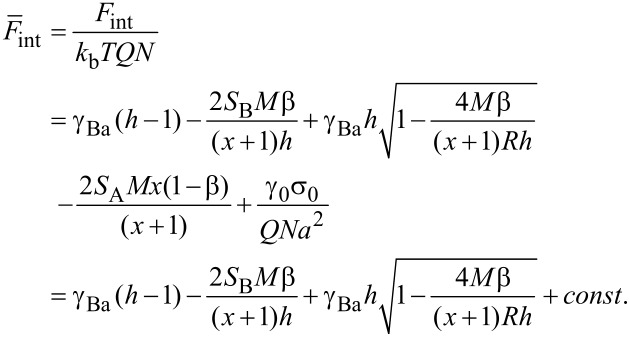


The elastic free energies of the core and the shell are calculated in a similar way to those of the disclike structure:

[14]
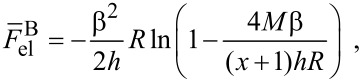


and

[15]
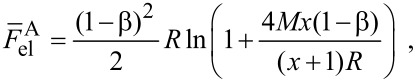


where the conditions in Equation 12 are used.

The confinement free energy has the same form as for the case of discs. Hence the total free energy of the torus-like micelles can be written as

[16]
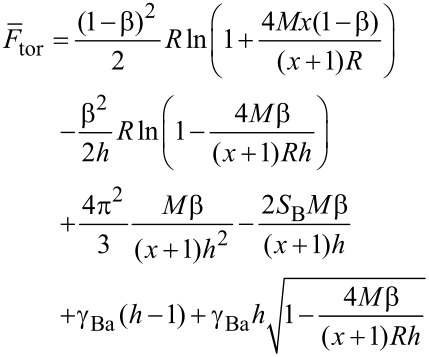


The equilibrium value is calculated by minimization with respect to the parameters *R* and *h*.

#### Stripes

**Figure 7:**
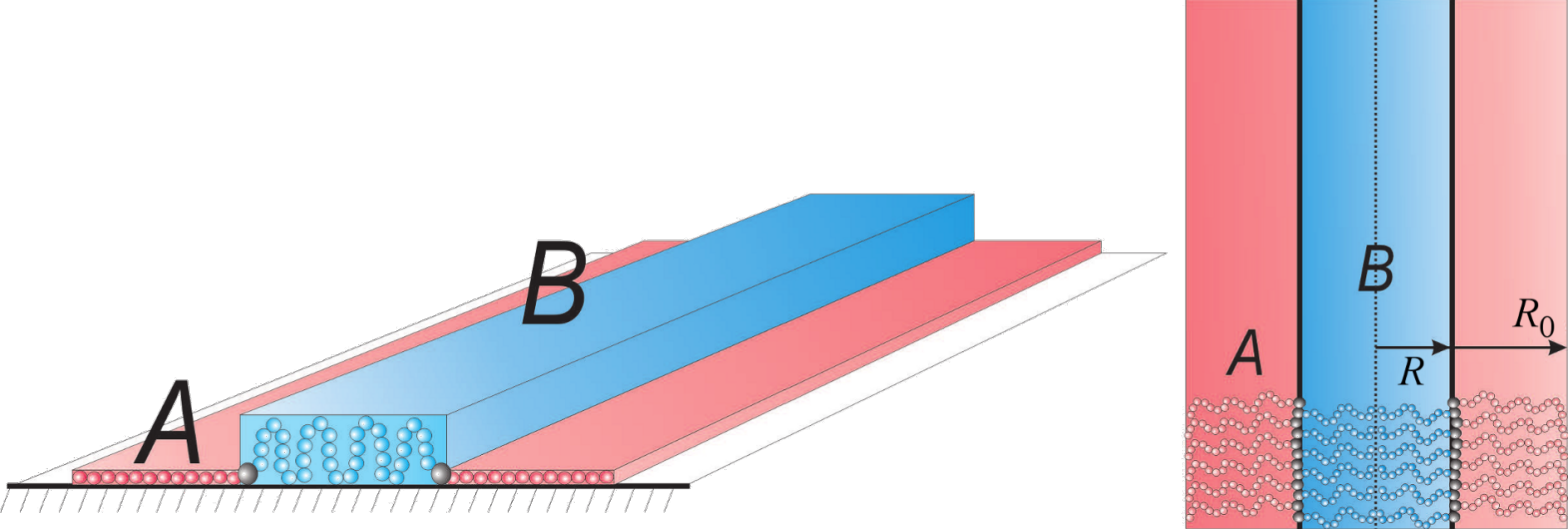
Schematic representation of a stripe-shaped structure.

If the value of the fraction of B side chains β increases, the torus-like structure becomes unfavorable and a stripes-like structure can be observed (Figure 7). The width of the A chains monolayer is 2(*R*_0_ − *R*). The B stripes have width 2*R* and thickness *h* (condition 1 ≤ *h* << *R* remains valid).

For this structure the total free energy has the form:

[17]
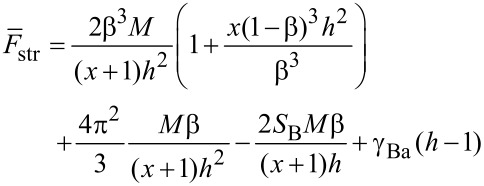


where the space-filling conditions are: 2*LRh* = *QN_B_M_B_* and 2*L*(*R*_0_ − *R*) = *QN_A_M_A_*, respectively. Here, *L* → ∞ is the length of the stripes. The first term in Equation 17 is the elastic free energy of the side chains, *N*_B_*R*^2^/*M*_B_ + *N_A_*(*R*_0_ − *R*)^2^/*M*_A_. Other terms are written similarly to the case of torus shaped micelles.

We can find conditions for the transition to a monomer thick structure (*h* = 1) for the stripes:


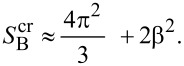


Thus, the completely two dimensional structure is stable if 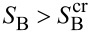.

#### Inverse Tori

**Figure 8:**
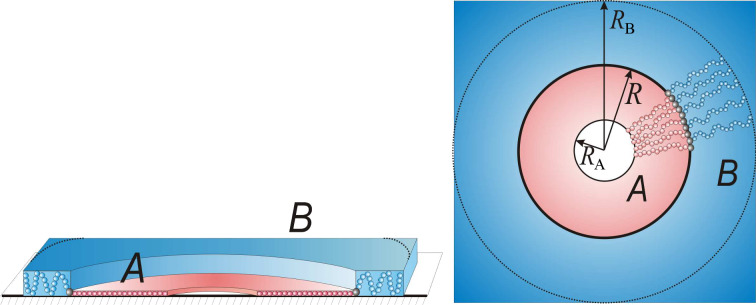
Schematic representation of an inverse torus-like structure.

In the case of a large asymmetry of the binary combs forming the film (*N*_B_ < *N*_A_), the inverse torus-shaped structure can be stable. Morphology of this structure is similar to that of the torus-shaped one, with the difference being that the inner part (core) of the micelles forms a monolayer of A type (Figure 8).

Similarly to the free energy of the conventional torus, Equation 16, the free energy of the inverse torus takes the form:

[18]
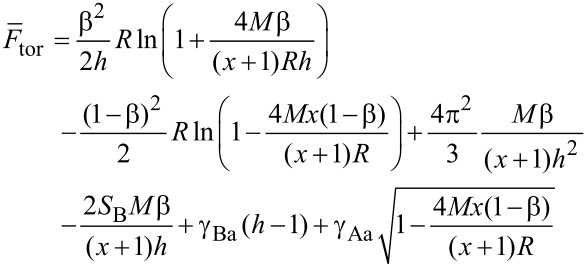


#### Holes

**Figure 9:**
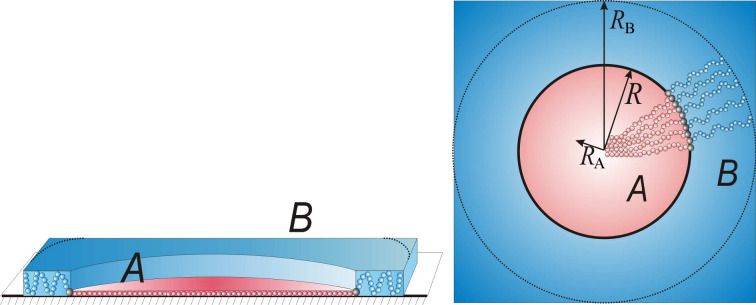
Schematic representation of “holes”

Finally, the last structure that can be observed in ultrathin film of the binary comb copolymers is the one inverse to the disk-like micelles (Figure 9). This structure is characterized by a disk-like, monomer thick core of the A side chains and by thickened corona of the B units.

The free energy of the “holes” takes a form similar to the case of disks, Equation 11:

[19]
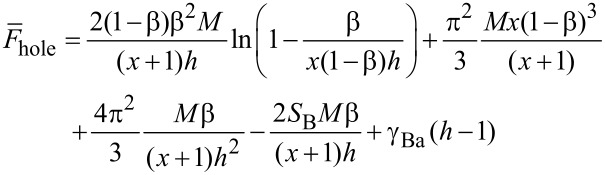


#### Phase diagrams

**Figure 10:**
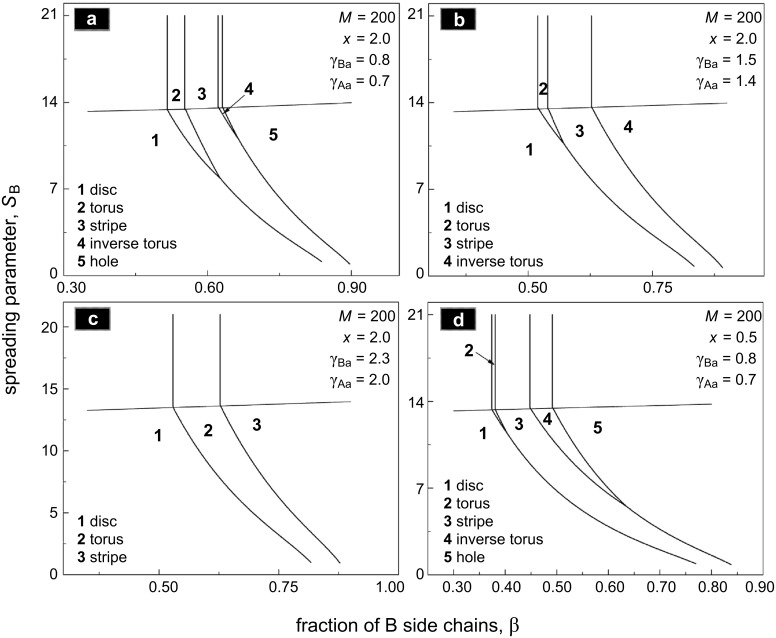
Phase diagram of the film in terms of the fraction of B side chains, β = *N*_B_/(*N*_A_ + *N*_B_), and the spreading parameter *S*_B_. The line 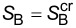 splits the regions of flat and prominent morphologies. The spreading parameter *S*_A_ satisfies the inequality 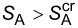 to ensure 2D conformation of A chains.

Phase diagrams in terms of the fraction of B side chains, β = *N*_B_/*N*, and of the spreading parameter, *S*_B_, are depicted in Figure 10. Boundaries between different nanostructures are determined from the conditions of equality of the free energies. The nearly horizontal line, 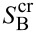, distinguishes the “landscape” of the film. Above the line (high values of *S*_B_), the film is completely flat consisting of 2D binary combs. Depending on interaction parameters, all analyzed morphologies can be stable. If β is small enough, a minor fraction of the B chains forms the core of the micelles in the film, i.e., disclike morphology. An increase of β may result in toruslike structure if the incompatibility of polymer B with the air (the surface tension coefficient γ_Ba_) is low enough. Indeed, the inner surface (line) of the torus possesses extra (in comparison with the disc) energy, which destabilizes the structure at high values of γ_Ba_. A further increase of β leads to the formation of a stripelike structure, which does not correspond to the symmetric composition β = 1/2. The reason for that is the value of the parameter *x* = *M*_A_/*M*_B_ = 2 (Figure 10a–c). One needs a higher fraction of short B chains on one side of the backbone to “equilibrate” the excluded volume of long A chains on the other side. The inverse toruslike structure is also stable only at relatively small values of the surface tension coefficient γ_Aa_ (Figure 10a). Finally, if β is high enough, the A blocks form the core of the micelles in the film.

Prominent nanostructures with elevated B domains are stable at 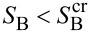. Here all the boundaries are shifted towards higher values of β. This effect can be explained by partial desorption (shrinkage) of the B chains, which is accompanied by the decrease of their lateral stretching. Therefore, in order to stabilize a certain structure at low values of *S*_B_, one needs to take molecules with higher β to increase the stretching of the B chains. Both direct and inverse toruslike structures disappear with the decrease of *S*_B_ at fixed values of γ_Ba_ and γ_Aa_. This behavior is also related to the energy of the inner surface of the torus: Decreasing *S*_B_ thickens the torus and increases the surface energy. The particular slope of the boundaries of the prominent morphologies allows us to conclude that the variation of the stickiness of one of the blocks in the film can lead to morphological transitions.

Changing of the parameter *x* from 2 to 0.5 (Figure 10a and Figure 10d) corresponds to the shortening of the A chains with respect to the B chains. In this case the whole phase diagram shifts towards lower values of β (one needs a smaller fraction of long side chains of B type to change the morphology).

**Figure 11:**
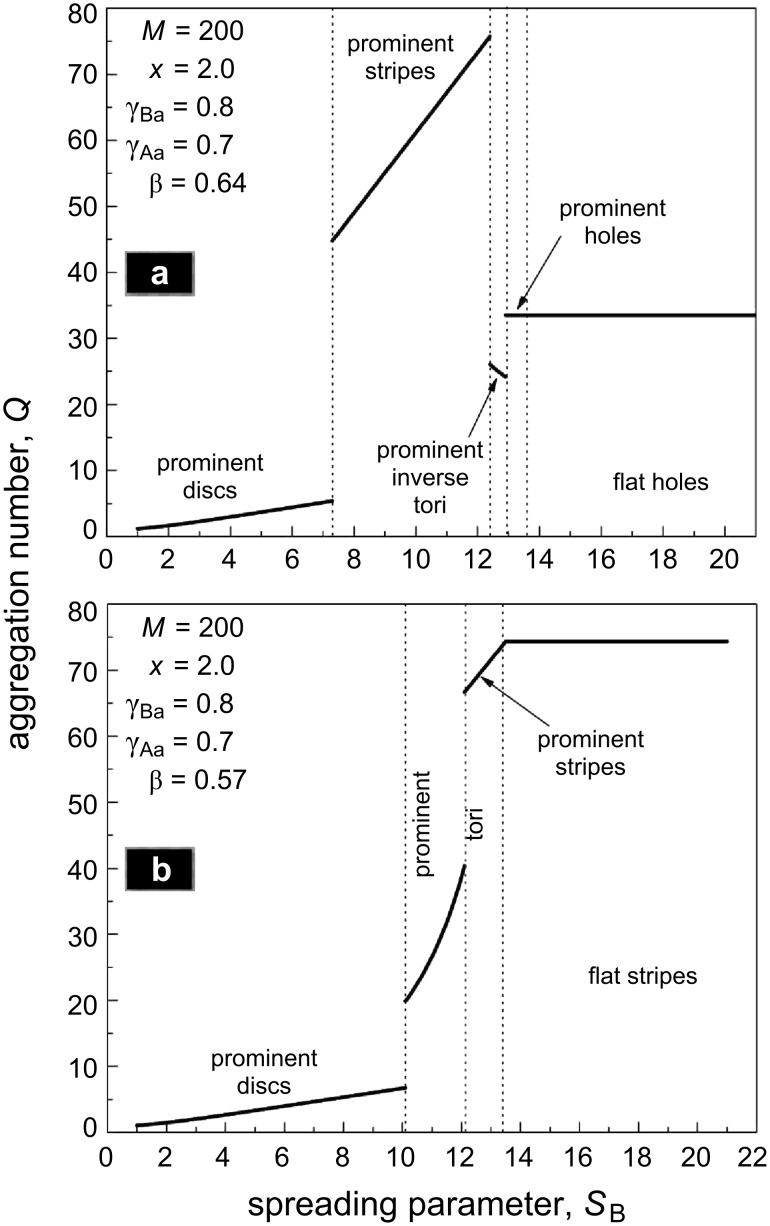
Aggregation number *Q* as a function of the spreading parameter *S*_B_ at different values of β: β = 0.64 (a) and 0.57 (b). The vertical lines split the regions of stability of various nanostructures.

Partial desorption of the B side chains influences not only the morphology but also the size and aggregation number of the micelles in the film (Figure 11). The increase in stretching of the B chains with *S*_B_ leads to a decrease in the value of the spontaneous curvature of each comblike molecule (the so-called energetic curvature [137]) and, hence, to the growth of the radius of the micelles (disc- or toruslike) and their aggregation number (Figure 11).

Direct and inverse toruslike nanostructures are characteristic structures of the binary combs and they are absent in the films formed by diblock copolymers [135–136]. This feature is related to the form of “building blocks” in self-organized films. The ability to form spontaneous curvature on the level of individual comblike macromolecules predefines stability of the toruslike structures.

## Conclusion

In conclusion, we can state that the improvement in procedures for the synthesis of comblike macromolecules makes it possible to prepare new classes of polymers with well-defined structures. In turn, this leads to the discovery of properties not typical of other types of molecules. Specifically, the effects of the strong extension of chains and the feasibility of controlling conformational properties on the surface are of indubitable interest for the creation of diverse molecular machines. Even though intensive studies of comblike polymers have been carried out for more than two decades, the question whether the conformational properties of such molecules in solution depend on structural parameters remains unsolved. However, considerable progress has been achieved in the study of polymers adsorbed on the surface.

At present, researchers have shifted their attention to comblike macromolecules of complex chemical structure. These systems are of the utmost interest for the discovery of new effects and for creating new materials.
